# Experimental Research on a New Mini-Channel Transcritical CO_2_ Heat Pump Gas Cooler

**DOI:** 10.3390/mi14051094

**Published:** 2023-05-22

**Authors:** Jiawei Jiang, Shiqiang Liang, Xiang Xu, Buze Chen, Zhixuan Shen, Chaohong Guo, Liqi Yu, Shuo Qin

**Affiliations:** 1 Institute of Engineering Thermophysics, Chinese Academy of Sciences, Beijing 100190, China; 2 Research Center of Fluid Machinery Engineering and Technology, Jiangsu University, Zhenjiang 212013, China; 3 School of Engineering Science, University of Chinese Academy of Sciences, Beijing 100049, China; 4 Jiangsu Zhongke Research Center for Clean Energy and Power, Lianyungang 222069, China

**Keywords:** transcritical carbon dioxide heat pump gas cooler, spiral heat exchanger, heat transfer, pressure drop

## Abstract

This paper presents the results of an experimental study on the heat transfer and pressure drop characteristics of a novel spiral plate mini-channel gas cooler designed for use with supercritical CO_2_. The CO_2_ channel of the mini-channel spiral plate gas cooler has a circular spiral cross-section with a radius of 1 mm, while the water channel has an elliptical cross-section spiral channel with a long axis of 2.5 mm and a short axis of 1.3 mm. The results show that increasing the mass flux of CO_2_ can effectively enhance the overall heat transfer coefficient when the water side mass flow rate is 0.175 kg·s^−1^ and the CO_2_ side pressure is 7.9 MPa. Increasing the inlet water temperature can also improve the overall heat transfer coefficient. The overall heat transfer coefficient is higher when the gas cooler is vertically oriented compared to horizontally oriented. A Matlab program was developed to verify that the correlation based on Zhang’s method has the highest accuracy. The study found a suitable heat transfer correlation for the new spiral plate mini-channel gas cooler through experimental research, which can provide a reference for future designs.

## 1. Introduction

**Figure 1:**
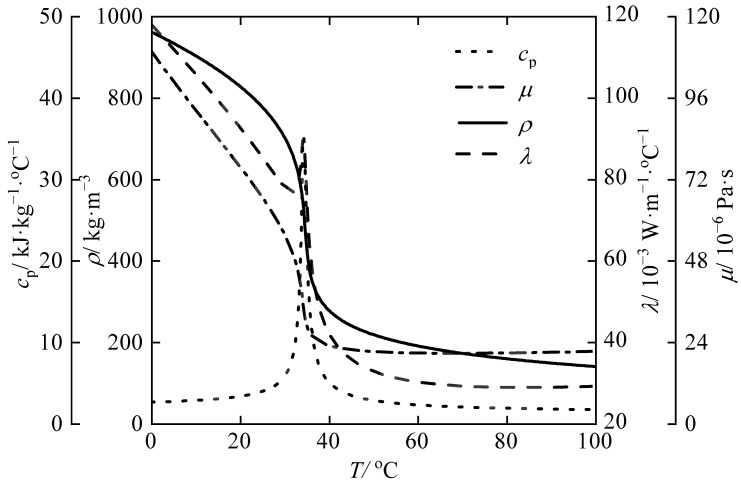
Physical properties of supercritical CO_2_ at *p* = 8 MPa.

It is widely known that the emergence of transcritical CO_2_ heat pump technology has led to increased attention to the flow and heat transfer characteristics of supercritical flu-ids. As a natural working fluid, CO_2_ has a lower critical point compared to other fluids, with a critical temperature of 31.1 °C and critical pressure of 7.38 MPa, making it easier for CO_2_ to reach its supercritical state. This has made the CO_2_ heat pump cycle very popular worldwide due to its excellent environmental protection and wide operating range [1]. Since fluids at supercritical state have unique properties, the heat transfer characteristics are more complicated and the design of heat exchanges is more challenging than that of conventional heat exchanges. In addition, considering the requirements of the operating pressure of 8–10 MPa and a small volume of the system, the heat exchanger calls for high strength and high compactness [2]. Therefore, it is critical to study the heat transfer and pressure drop characteristics of supercritical CO_2_ in compact heat exchangers. Figure 1 shows the variation of specific heat, thermal conductivity, viscosity, and density of CO_2_ with the temperature at the pressure of 8 MPa. In the early days of research about heat transfer of supercritical CO_2_, considerable efforts have been made to study the convective heat transfer characteristics of supercritical CO_2_ in a single tube or simple types of heat exchangers [3,4,5,6]. Research on the heat transfer characteristics of supercritical CO_2_ in spiral channels is currently limited. However, with the rapid development of processing and manufacturing technologies such as diffusion welding and brazing, several types of compact heat exchangers have been used in supercritical CO_2_ facilities. The printed circuit heat exchanger (PCHE) is considered suitable for recuperators due to its small channels and excellent pressure and temperature tolerance. Some scholars also investigate the heat transfer between CO_2_ and water in PCHEs. It has been noted that the high cost of PCHEs can make them less competitive as a gas cooler option. As an alternative, a microtube shell-and-tube heat exchanger (MSTE) was proposed. However, it has a small shell-side space, being prone to scale, difficult to clean, and not suitable for use as a CO_2_ heat pump gas cooler. In transcritical CO_2_ heat pumps, the water side of the gas coolers can be prone to fouling during long-term operations, but spiral plate heat exchangers (SPHEs) can ad-dress the issue. SPHEs’ unique geometry eliminates stagnant areas in the channel, resulting in a low fouling tendency. Therefore, SPHEs may be a more suitable option for CO_2_ heat pump gas coolers than MSTEs or PCHEs. The spiral plate heat exchanger has a long history dating back to 1930, when it was first proposed by Rosenblad in Sweden. Over the years, it has undergone numerous improvements and innovations. Coons et al. [7] tested five different SPHEs and collected data on pressure, temperature, velocity, mass flow rate, and pressure drop for the heating and cooling of oil, water, brine, and steam. For spiral exchangers operating under laminar flow conditions, the average Nusselt number correlation was suggested. Baird et al. [8] developed a correlation for calculating the heat transfer coefficient for a Rosenblad SPHE. Water was used as the working fluid for both ducts. They assumed that the temperature change would be linear along the spiral length and used the mean spiral radius as the radius at the mean spiral length. Tangri and Jayaraman [9] tested the applicability of the Dittus–Boelter equation for predicting heat transfer in an SPHE. The overall heat transfer coefficient was found to decrease with an increase in the spiral radius. Buonopane and Troupe [10] derived the equations to describe the thermal performance of SPHEs by making energy balance on a differential wedge element of an SPHE. Heat transfer correlations were developed using a modified Wilson plot technique. Zhang et al. [11] developed a computational method for the thermal design of an SPHE. Bes and Roetzel [12,13,14] analyzed the thermal performance of a spiral heat exchanger and developed an analytical method to evaluate fluid temperature variation in a countercurrent spiral heat transfer. Shirazi et al. [15] developed a new algorithm for spiral plate heat exchangers; the algorithm was optimized in terms of geometric aspect ratio, pressure drop, and total cost. The relative heat rate capacity per volume of the newly designed spiral plate heat exchanger reached up to 54%, compared to other designs. Rajavel et al. [16] tested a spiral plate heat exchanger, and a new correlation based on the experimental data was given for practical applications. A numerical method was proposed by Garcia et al. [17] to estimate the temperature distribution and overall heat transfer coefficients based on the flow rates and temperatures at inlet and outlet. The first significant numerical study was published by Devois et al. [18]. They produced a thermal model of the heat exchanges in both steady-state and time-dependent cases with a 2D spiral geometry, allowing computation with different materials, forced convective heat transfer models in turbulent flow, and geometrical parameters options. Nguyen et al. [19,20] developed a numerical method to investigate the heat transfer performance of a spiral heat exchanger. Many researchers have focused mainly on experimental studies to validate their research results and numerical methods, with particular emphasis on designing and optimizing spiral plate heat exchangers. However, there are relatively few examples of innovative designs on the original structure. The heat transfer correlation proposed by the researchers is mostly based on water, and little research has been carried out on carbon dioxide. In addition, the forms of heat transfer correlation vary among researchers. Therefore, further research is needed to establish more accurate and reliable heat transfer correlations for spiral plate mini-channel gas coolers. Furthermore, there has been no experimental study to date on the heat transfer and pressure drop characteristics of the new spiral plate mini-channel gas cooler. Further research is therefore needed to better understand these characteristics and optimize the design of this type of heat exchanger. This study described in this paper builds on previous research that demonstrated the feasibility of gas coolers using numerical simulations [21]. The authors produced a physical prototype using diffusion bonding and conducted experiments to analyze internal heat transfer patterns. Through these experiments, the authors were able to develop accurate heat transfer correlations and pressure drop calculation methods. The traditional method of calculating pressure drops for spiral plate heat exchangers is no longer applicable, as previous studies have been based on hot and cold water, and numerical simulation methods are not universally applicable. The authors’ work represents an important contribution to the field, providing new insights into the performance characteristics of spiral plate heat exchangers with gas cooling applications. By developing more accurate heat transfer correlations and pressure drop calculation methods, the authors have laid the foundation for future research aimed at optimizing the design and operation of these systems.

In this study, a 10 kW supercritical CO_2_ heat exchanger experimental platform is built, and a new spiral plate mini-channel gas cooler is designed and processed to have a better understanding of the cooling heat transfer characteristics of supercritical CO_2_ in the new spiral plate mini-channel gas cooler. In Section 2, the experimental system and gas cooler structure are described. In Section 3, the effects of different variables on heat transfer, the accuracy of different heat transfer correlations, and the accuracy of pressure drop calculation methods are analyzed. The findings are provided in Section 4.

## 2. Experiment Setup

### 2.1. Experiment System

**Figure 2:**
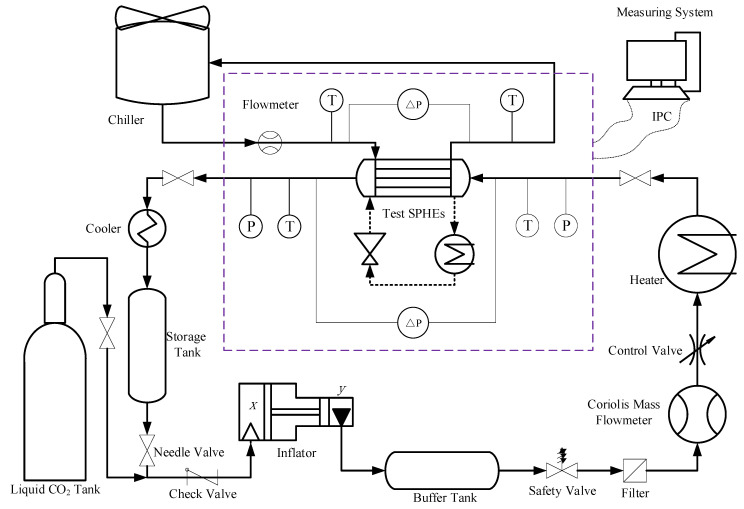
Schematic of experimental system.

**Figure 3:**
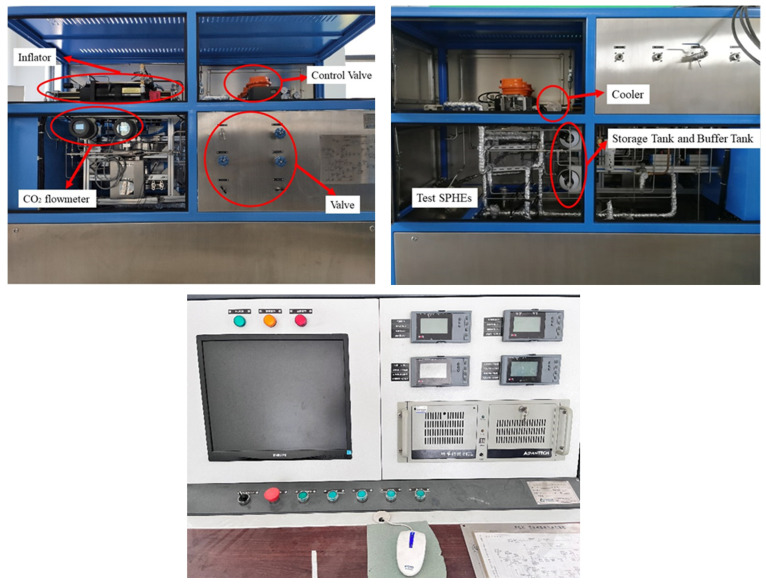
Test platform and console.

**Figure 4:**
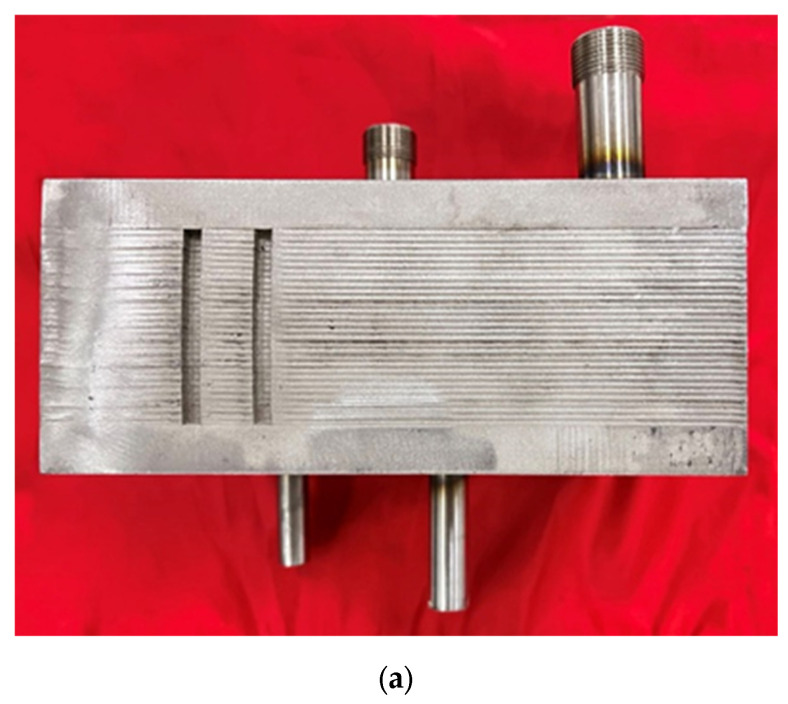

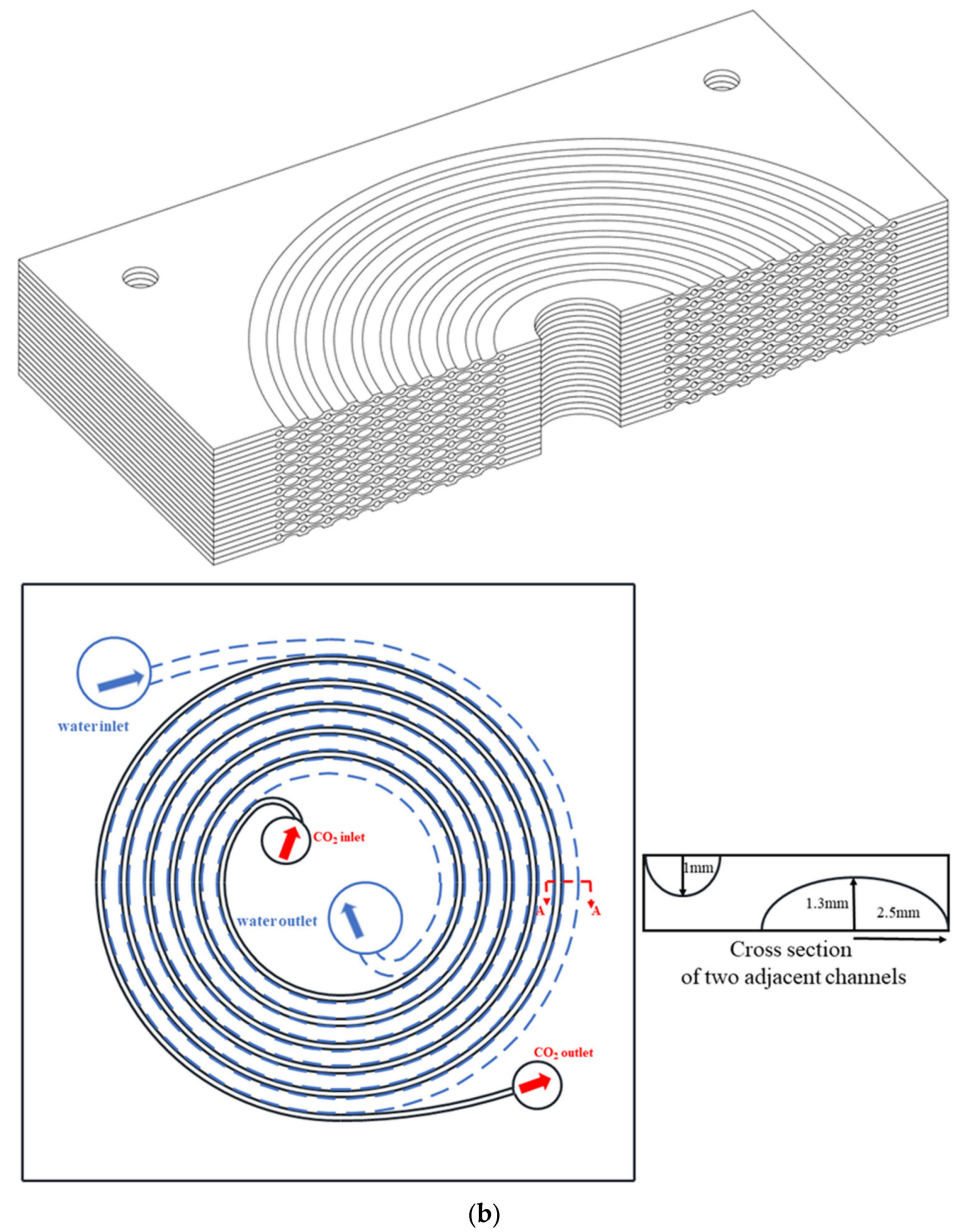
(**a**) Object-in-kind; (**b**) overall schematic.

Figure 2 shows a schematic diagram of the experiment system, and Figure 3 shows a physical diagram of the experiment system. The system consists of various components, including a compressor, liquid CO_2_ storage tank, buffer tank, chiller, cooler, measuring sensors, electric heater, data acquisition module, and control console. The compressor used in this system is a plunger pump that can be cooled by compressed air at room temperature. This approach allows CO_2_ pressurization under a variety of conditions without any concern for seal failure due to overheating of the pump chamber. Carbon dioxide is directly heated by the electric heater. The outlet pressure of the compressor can reach 25 MPa, and the maximum flow rate is 0.05 kg·s^−1^. The maximum heating power of the preheater is 25 kW. In addition to the various components described above, the experimental platform is also equipped with a remote console. This console can remotely control the compressor, pneumatic valves, electric heater heating power, and data monitoring and recording via a computer. Overall, this feature adds another layer of safety to the experimental setup, ensuring that researchers can conduct experiments effectively and without risk to their personal safety. The object-in-kind of the new spiral plate mini-channel gas cooler is shown in Figure 4. The overall schematic is shown in Figure 4b, and the important dimensions of the gas cooler are listed in Table 1. Information on experimental equipment is summarized in Table 2.

During the experiment, liquid CO_2_ is initially filled into the entire system loop from the storage tank. Once the liquid CO_2_ filling is complete, the filling circuit valve is closed, and the main circulation circuit valve is opened to circulate CO_2_ using the booster pump. To heat and pressurize the system, an electric heater is used, while cooling water is introduced into the terminal cooler to ensure that the temperature at the pump inlet does not exceed the limit. In addition, a buffer tank after the compressor can effectively eliminate flow fluctuations caused by the reciprocating movement of the pump, making the experimental flow more stable and the results more accurate.

### 2.2. Date Reduction

**Figure 5:**
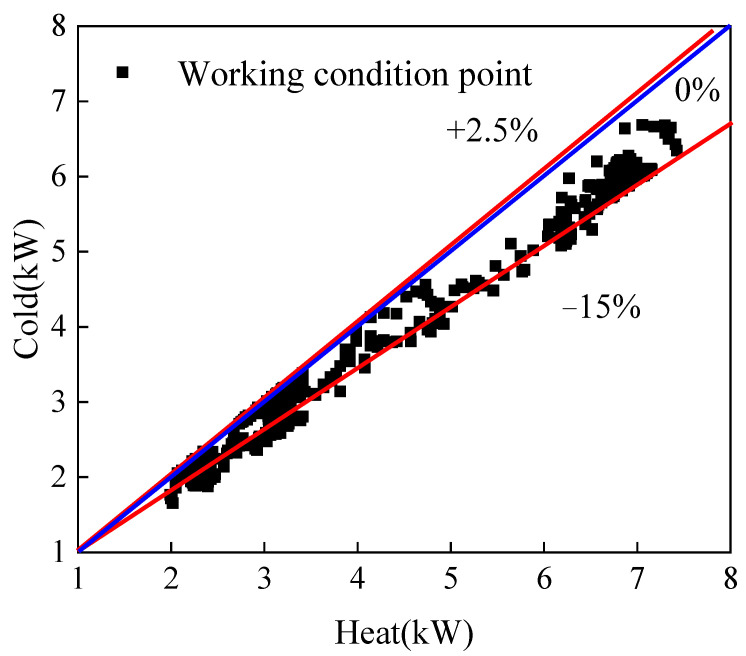
Heat balance diagram under different working conditions. The blue line is the heat balance line, the red line is the error line.

The experimental system used in this study was designed to test the performance of a gas cooler when placed horizontally and vertically. To ensure the accuracy of the results, efforts were made to minimize heat dissipation from the experimental system to the environment. Experimental data revealed that there was a difference of no more than 15% be-tween the heat absorbed by the cold fluid and the heat released by the hot fluid that is acceptable for engineering applications. Figure 5 shows the heat exchange error under different working conditions. In most of the working conditions tested, the heat release from the hot side of the gas cooler was greater than the heat absorption from the cold side. It is possible that the experimental conditions were constantly changing, resulting in some of the heat being absorbed by the solid wall. As a result, the temperature between the solid and the two fluids did not reach thermal equilibrium. These findings suggest that the gas cooler is effective in cooling and could be used in a variety of engineering applications. The overall heat exchange of the gas cooler was calculated using the mass flow rate of carbon dioxide and enthalpy at the inlet and outlet points, as shown:

(1) $$Q_{{\mathrm{CO}}_{2}}={\dot{m}}_{{\mathrm{CO}}_{2}}(h_{{\mathrm{CO}}_{2},\mathrm{in}}-h_{{\mathrm{CO}}_{2},\mathrm{out}})$$

The temperature difference between the cold and the hot fluid can be calculated using the logarithmic mean temperature difference, as follows:

(2) $$\Delta T_{\mathrm{LMTD}}=\frac{\max(\Delta T_{1},\Delta T_{2})-\min(\Delta T_{1},\Delta T_{2})}{\ln(\max(\Delta T_{1},\Delta T_{2})/\min(\Delta T_{1},\Delta T_{2}))}$$

As the heat transfer areas on the sides are not the same, the heat transfer area on the carbon dioxide side is used as the basis for calculating the total heat transfer coefficient for the spiral plate mini-channel gas cooler, as shown:

(3) $$U=\frac{1}{\frac{1}{U_{{\mathrm{CO}}_{2}}}+\frac{\delta }{\lambda }+\frac{A_{{\mathrm{CO}}_{2}}}{U_{water}A_{water}}}$$

The total heat transfer coefficient can also be calculated according to the following formula:

(4) $$Q=UA\Delta T_{LMTD}$$

The heat transfer coefficient of water can be calculated using the empirical correlation as follows:

(5) Uwater=0.05Re0.6459Pr0.4μbμw0.14λd

Multiple correlations were used on the CO_2_ side for comparison, including Mcadams, Inagaki, Morimoto, Zhang, and Minton, as shown in Table 3 [11,12,13,14,15,16,17,18,19,20,21,22,23,24,25,26]. The traditional flow resistance calculation method for a spiral plate heat exchanger is mainly based on the Sauder equation, which was derived experimentally to calculate pressure loss. How-ever, since conventional spiral plate heat exchangers have a fixed column distance, these equations cannot be applied. Therefore, a more accurate calculation method needs to be found. An adjusted formula was introduced incorporating the number of spiral turns and the length, as given in [27,28]:

(6) $$\Delta P=\frac{1.5G^{2}N}{2}(\frac{1}{\rho_{i}}+\frac{1}{\rho_{o}})+\frac{fLG^{2}}{2d\rho_{m}}+G^{2}(\frac{1}{\rho_{o}}-\frac{1}{\rho_{i}})+\frac{\rho_{m}v^{2}}{2}k$$

(7) 1f=3.48−1.7372lnεa−16.2426Relnε/a1.10986.0983+7.149Re0.8981

where ε is the surface roughness, and a is half of the hydraulic diameter.

**Figure 6:**
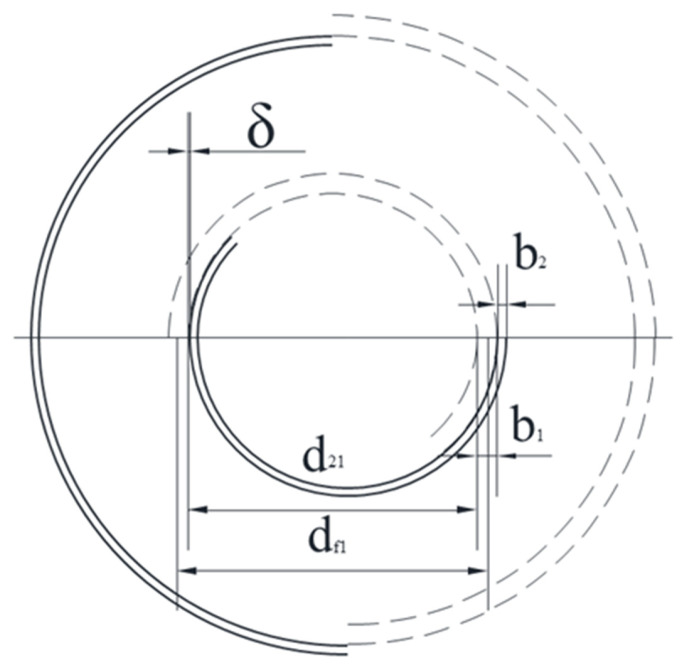
Scheme for calculation of the outer diameter.

The heat transfer and hydraulic characteristics of fluids within spiral channels can be predicted based on the geometric parameters of spiral channels. Figure 6 shows the primary geometric parameters of a counterflow spiral plate heat exchanger, including channel width, channel spacing, initial spiral diameter, outer spiral diameter, and initial spiral channel diameter, which can be calculated using Equation (8):

(8) $$d_{f1}=d_{21}+b_{1}+\delta$$

In the equation, *d*_21_ represents the inner diameter of the water side channel, and the outer diameter of the spiral channel can be calculated using Equation (9):

(9) $$D_{s}=\sqrt{d_{f1}^{2}+1.28L(b_{1}+b_{2}+2\delta )}$$

The number of spiral turns can be obtained from the following equation.

(10) $$N=\frac{-(d_{f1}-\frac{b_{1}+b_{2}+2\delta }{2})+\sqrt{{\left(d_{f1}-\frac{b_{1}+b_{2}+2\delta }{2}\right)}^{2}+\frac{4L(b_{1}+b_{2}+2\delta )}{\pi }}}{2(b_{1}+b_{2}+2\delta )}$$

The hydraulic diameter is calculated by the following equation.

(11) $$d=\frac{4\times \mathrm{cross}\;\mathrm{section}}{\mathrm{wetted}\;\mathrm{perimeter}}$$

The Reynolds number is calculated as follows:

(12) Re=mdμA

The Prandtl number is calculated as follows:

(13) $$\mathrm{Pr}=\frac{\mu c_{p}}{\lambda }$$

**Figure 7:**
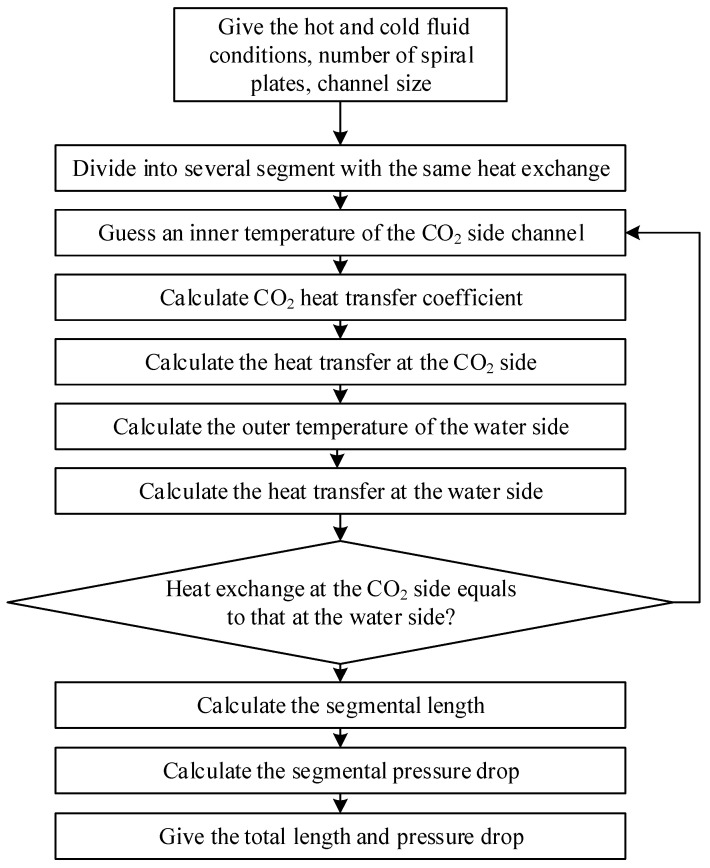
Spiral plate mini-channel gas cooler design program.

For the supercritical CO_2_-water experiment, it is not suitable to directly analyze the heat transfer coefficient using the logarithmic mean temperature difference method due to the large change in the physical properties of supercritical CO_2_. The complex manufacturing technique also makes it difficult to measure the temperature of the tube wall directly. As a result, a segmented calculation program is developed based on the existing correlations of the CO_2_ heat transfer coefficient to predict heat transfer and pressure drop between supercritical CO_2_ and water in the spiral plate mini-channel gas cooler, as shown in Figure 7. The gas cooler model is based on the principles of energy conservation, and establishes a one-dimensional submodel with variable physical properties of sCO_2_. The model assumes that the temperature of the CO_2_ inner wall can be used to calculate the heat flux on both the CO_2_ and the water sides, and from there the heat transfer coefficients on both sides can be calculated. The model then iteratively calculates until the difference between the CO_2_ and the water side heat exchange is less than 0.1% of the CO_2_ side heat exchange.

However, there are some assumptions made in this model.

(1) Axial thermal conductivity and ambient heat dissipation are ignored;
(2) The specific heat, density, and thermal conductivity of the metal wall are constants;
(3) There is a fully developed turbulent flow without entrance and exit effects;
(4) A uniform flow distribution in each channel of the plate is assumed by the model.

### 2.3. Uncertainty Analysis

In the supercritical CO_2_–water heat exchange experiment, the uncertainty in the water–side heat exchange mainly arises from the error in the flow and temperature measurements. The calculated error resulting from the temperature measurement is expressed using the following formula, which represents the difference between inlet and outlet temperatures [29]:

(14) $$\frac{\delta \Delta T_{\mathrm{water}}}{\Delta T_{\mathrm{water}}}=\frac{\left|\delta T_{\mathrm{water},\mathrm{in}}\right|+\left|\delta T_{\mathrm{water},\mathrm{out}}\right|}{T_{\mathrm{water},\mathrm{in}}-T_{\mathrm{water},\mathrm{out}}}$$

where $\delta T_{\mathrm{water}}$ is determined to be ±0.2 °C. The calculation error of the heat exchange is as follows:

(15) $$\frac{\delta Q_{\mathrm{water}}}{Q_{\mathrm{water}}}=\sqrt{{\left(\frac{\delta {\dot{m}}_{\mathrm{water}}}{{\dot{m}}_{\mathrm{water}}}\right)}^{2}+{\left(\frac{\delta \Delta T_{\mathrm{water}}}{\Delta T_{\mathrm{water}}}\right)}^{2}}$$

The error in measuring the mass flow rate for water is ±0.5%. The error of the logarithmic mean temperature difference is calculated by the following formula:

(16) $$\frac{\delta \Delta T_{\mathrm{LMTD}}}{\Delta T_{\mathrm{LMTD}}}=\frac{\delta T_{{\mathrm{CO}}_{2},\mathrm{in}}+\delta T_{{\mathrm{CO}}_{2},\mathrm{out}}+\delta T_{\mathrm{water},\mathrm{in}}+\delta T_{\mathrm{water},\mathrm{out}}}{\max(\Delta T_{1},\Delta T_{2})-\min(\Delta T_{1},\Delta T_{2})}$$

The calculation of the CO_2_ heat exchange is based on the calculation of the mass flow rate of CO_2_ and the enthalpy of inlet and outlet, and its uncertainty is calculated as follows:

(17) $$\left|\frac{\delta Q_{{\mathrm{CO}}_{2}}}{Q_{{\mathrm{CO}}_{2}}}\right|=\sqrt{{\left(\frac{\delta {\dot{m}}_{{\mathrm{CO}}_{2}}}{{\dot{m}}_{{\mathrm{CO}}_{2}}}\right)}^{2}+{\left(\frac{\left|\delta H_{\mathrm{in}}\right|+\left|\delta H_{\mathrm{out}}\right|}{H_{\mathrm{in}}-H_{\mathrm{out}}}\right)}^{2}}$$

The mass flow rate error is ±0.2% from a Coriolis mass flowmeter, and the calculation of CO_2_ inlet and outlet enthalpy is mainly based on the experimentally measured CO_2_ inlet and outlet temperature and pressure. Therefore, the error mainly includes the mass flow rate, temperature, and pressure of inlet and outlet, which is calculated as follows:

(18) $$\frac{\delta H}{H}=\frac{H(T\pm \delta T,P\pm \delta P)-H(T,P)}{H(T,P)}$$

Under the experimental pressure and temperature range, the error of total heat transferred by CO_2_ is within 10%.

## 3. Results and Discussion

### 3.1. Effect of Inlet Mass Flux of CO_2_ on Heat Transfer

**Figure 8:**
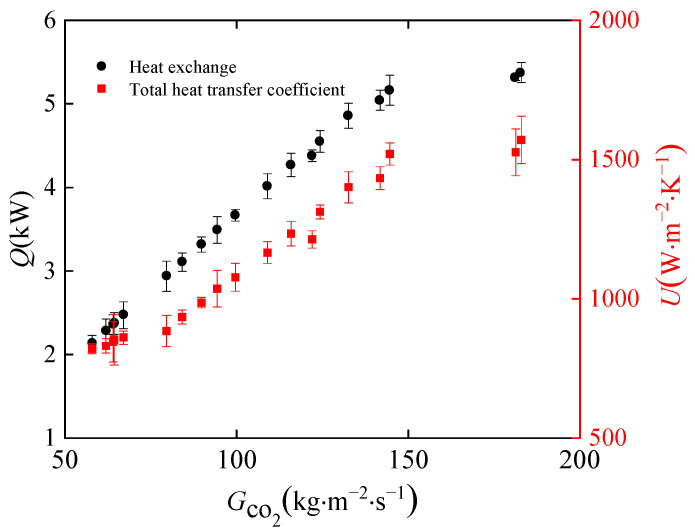
Effect of heat exchange and heat transfer coefficient of CO_2_ at different mass flux.

In this section, the effects of CO_2_ mass fluxes on heat transfer characteristics are discussed. Figure 8 shows the variation in the total heat transfer coefficient and heat exchange for different mass fluxes of CO_2_ in the gas cooler. The mass flow rate of water is 0.175 kg·s^−1^, and the pressure of CO_2_ is 7.9 MPa. The maximum CO_2_ mass flow rate is 0.03 kg·s^−1^. As the CO_2_ mass flux increased from 57 kg·m^−2^·s^−1^ to 182 kg·m^−2^·s^−1^, the heat exchange and total heat transfer coefficient increased in an essentially linear trend, and the heat exchange increased by 3 kW. With a mass flux of CO_2_ of 182 kg·m^−2^·s^−1^, the total heat transfer coefficient reaches 1570 W·m^−2^·K^−1^. The total heat transfer coefficient is mainly influenced by the heat transfer coefficient of CO_2_ and water. The larger the mass flux of CO_2_, the stronger the turbulence of the fluid in the channel, and the higher the heat transfer coefficient of CO_2_. Increasing the mass flux of CO_2_ can significantly improve the total heat transfer coefficient. Increasing the heat transfer coefficient on either side is beneficial to the increase of the total heat transfer coefficient.

### 3.2. Effect of Placement Style of CO_2_ on Heat Transfer

**Figure 9:**
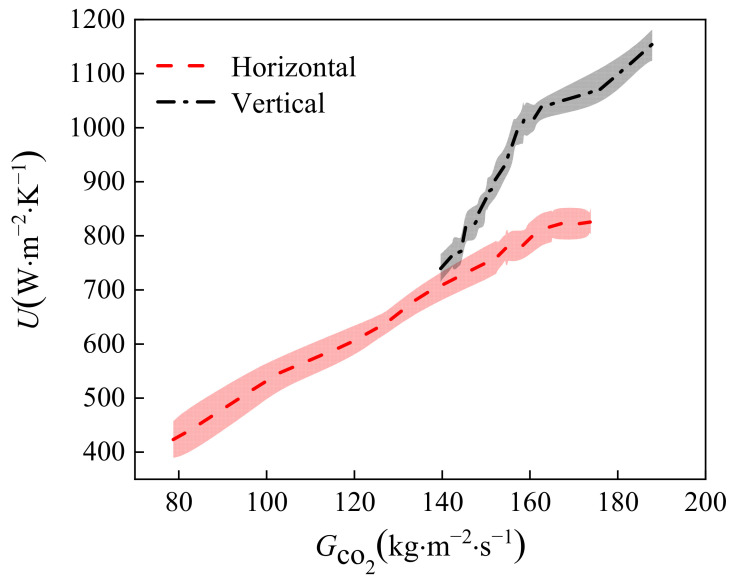
Variation of the heat transfer coefficient of CO_2_ at different placement style.

Figure 9 shows the variation in the total heat transfer coefficient of the gas cooler in the case of parallel flow when placed horizontally and vertically. The mass flow rate of water is 0.175 kg·s^−1^, and the CO_2_ pressure is 7.9 MPa. It can be observed that when placed vertically, the total heat transfer coefficient is greater than in the horizontal placement case because the water affected by gravity accumulates in the lower part of the gas cooler. CO_2_ enters the middle of the gas cooler and is in full contact with cooling water, resulting in a higher heat transfer coefficient than in horizontal placement. When placed vertically, high-temperature CO_2_ and water flow into the gas cooler at the same time, gravity accelerates the speed of the water flow to remove the heat gained faster, and the cycle continues.

### 3.3. Effect of Inlet Temperature of Water on Heat Transfer

**Figure 10:**
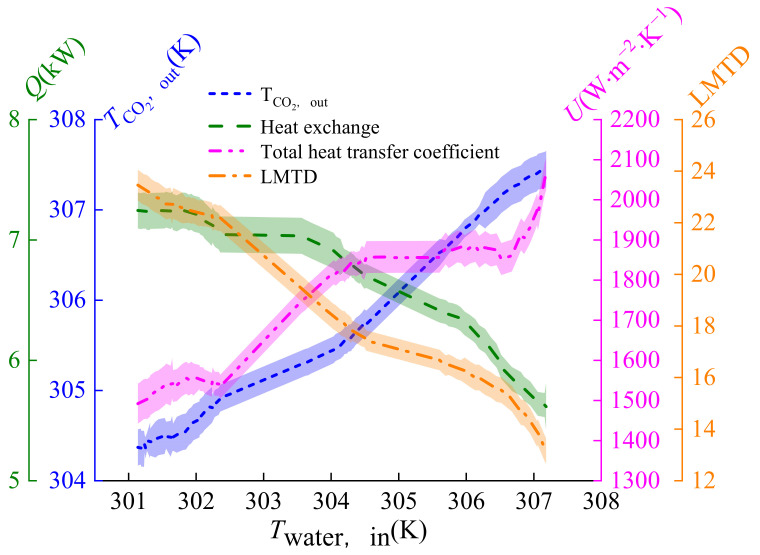
Effect of inlet temperature on heat transfer coefficient of CO_2_.

Figure 10 shows the effect of inlet water temperature on the heat transfer coefficient in the 301~308 K range. The heat exchange of the gas cooler decreases by 1.6 kW. The total heat transfer coefficient shows an increasing trend, indicating that the total heat transfer coefficient can be improved by increasing the inlet water temperature. The main reason is that the temperature reduction of carbon dioxide is greater than the temperature reduction of the inlet water. This is good for maintaining the temperature slippage between CO_2_ and water, making the heat exchange between water and CO_2_ sufficient. The logarithmic mean temperature difference reduction is greater than the heat exchange reduction, thus improving the total heat transfer coefficient. As the inlet water temperature increases, the total heat transfer coefficient increases from 1489 W·m^−2^·K^−1^ to 2063 W·m^−2^·K^−1^, and the CO_2_ outlet temperature is far from the critical point, leading to a in heat transfer.

### 3.4. Supercritical CO_2_–Water Experiment

In this section, heat transfer between supercritical CO_2_ and cooling water is examined experimentally. The results of heat transfer of CO_2_ and water in the spiral plate mini-channel gas cooler under different working conditions are presented in Table 4. CO_2_ pressure ranges from 7.5 MPa to 8.5 MPa, and water temperature ranges from 26 °C to 31 °C. Based on five supercritical CO_2_ correlations of the heat transfer coefficient, as shown in Table 3, a computational procedure for the spiral plate mini-channel gas cooler is developed. In addition, the CO_2_ outlet temperature, total length, CO_2_, and water pressure drop are obtained from the calculation, and the comparison between the experimental results and calculation results is discussed.

**Figure 11:**
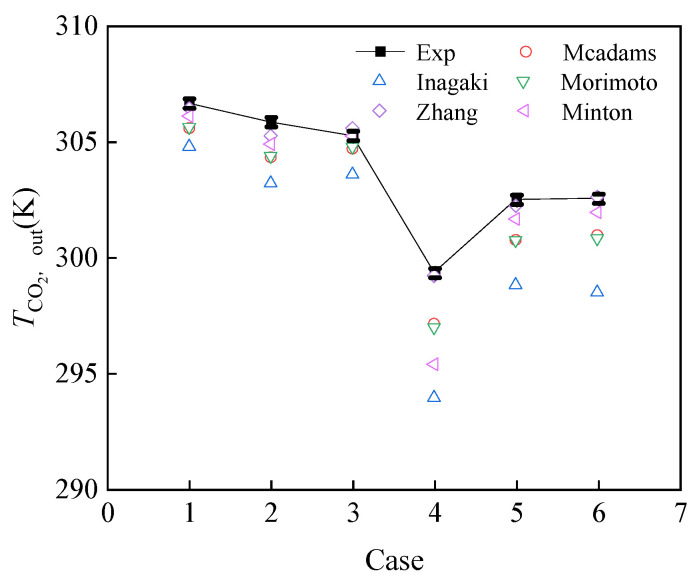
The comparison between predicted $T_{{\mathrm{CO}}_{2},\mathrm{out}}$ and experimental $T_{{\mathrm{CO}}_{2},\mathrm{out}}$.

Figure 11 compares the calculated CO_2_ outlet temperature of the program with the experimental results under different conditions. The outlet temperatures calculated from Zhang’s correlation show the best agreement with the experimental results among the five correlations. Most errors are within 1 °C. Inagaki’s correlation underestimates the CO_2_ outlet temperature in all cases and shows larger errors of 3–5 °C. Morimoto’s correlation also shows greater errors.

**Figure 12:**
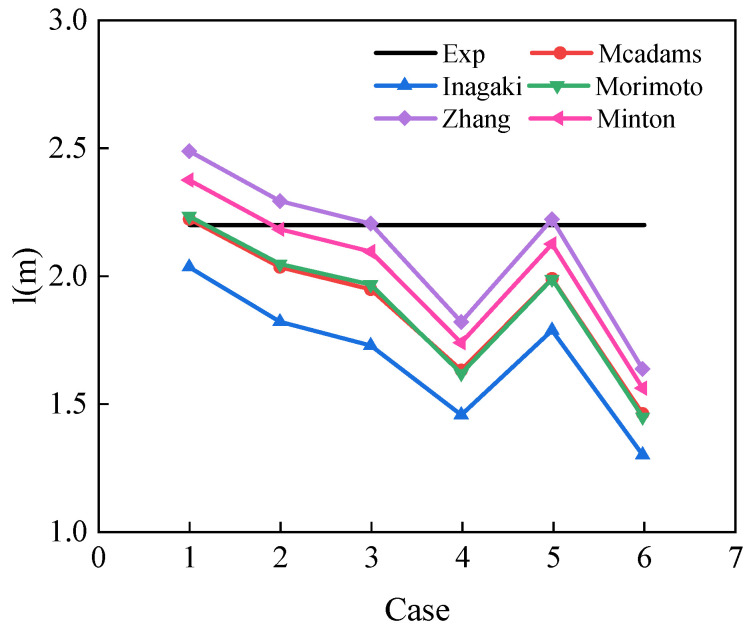
The predicted *l* under different cases.

Figure 12 shows the channel lengths calculated by different correlations under different working conditions, and the accuracy of each correlation for engineering calculation can be assessed by comparing the actual length of the spiral plate mini-channel gas cooler with the length calculated by the program. The design length of the existing correlations is longer than the actual length, i.e., 2.2 m. Zhang’s and Minton’s correlations overestimate the design length under high mass flow rate conditions. Zhang’s correlation has the least deviation and Minton’s correlation is also acceptable because it accounts for the effect of variable specific heat capacity of CO_2_ at different pressures. However, Inagaki’s correlation shows the highest discrepancy, indicating that it overestimates the heat exchange between CO_2_ and water. Mcadams and Morimoto’s correlations yield similar design values for each case, but with different coefficients, and both methods show similarities in predicting rotational flow. All the correlations show a decrease in design values with a decrease in mass flux. Moreover, the prediction errors of all correlations are greater at lower inlet water temperature and higher CO_2_ pressure. Inagaki’s correlation exhibits an unacceptable prediction error of up to 40% at 8.5 MPa. This is mainly because Inagaki studied a spiral coil heat exchanger rather than a spiral plate heat exchanger. Based on the comprehensive analysis, Zhang’s correlation is considered to provide the most accurate results in one-dimensional engineering design.

**Figure 13:**
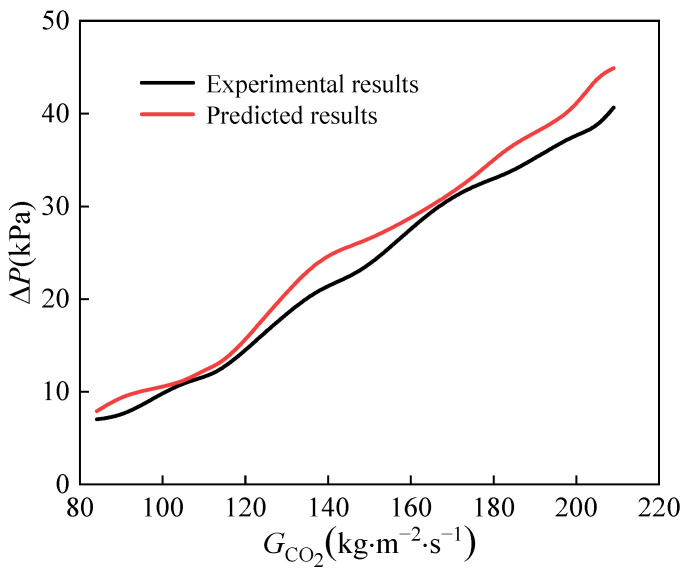
The comparison between predicted $\Delta p_{CO_{2}}$ and experimental $\Delta p_{CO_{2}}$.

**Figure 14:**
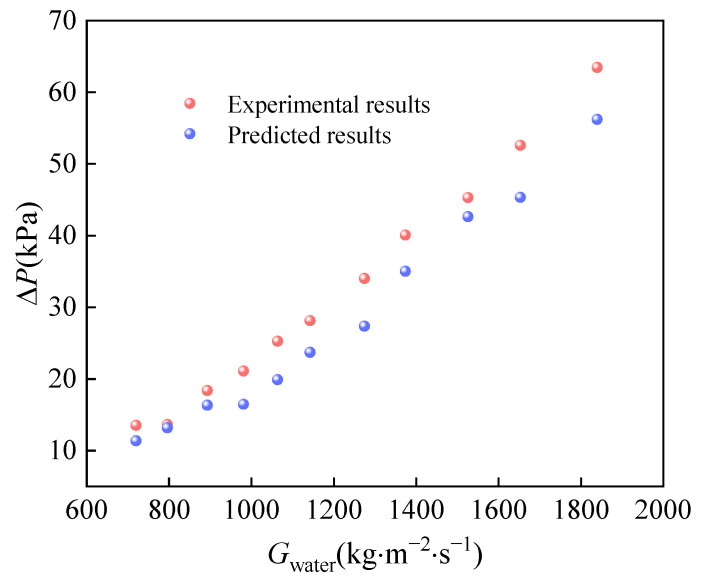
The comparison between predicted $\Delta p_{water}$ and experimental $\Delta p_{water}$.

Figure 13 and Figure 14 illustrate the comparison between predicted and experimental pressure drops under various mass fluxes of CO_2_ and water. It is evident that the experimental pressure drop is lower than predicted due to the lower viscosity of CO_2_ at supercritical pressure. The error between predicted and experimental pressure drops on both carbon dioxide and water is small. Maximum error between predicted and experimental carbon dioxide pressure drops is only 4 kPa. The experimental pressure drops on both sides are higher than those of the conventional heat exchanger; therefore, we must consider the balance of heat transfer and pressure drop in the subsequent design.

## 4. Conclusions

In this study, an experimental analysis of the heat transfer between CO_2_ and water in the spiral plate mini-channel gas cooler is made. The key findings are outlined as follows:

(1) Increasing the CO_2_ mass flux intensifies the turbulence within the channel, enhances heat transfer, and boosts the heat transfer coefficient of the gas cooler. The heat exchange and the total heat transfer coefficient show an almost linear increase within the range of 57 kg·m^−2^·s^−1^ to 182 kg·m^−2^·s^−1^ for the CO_2_ mass flux.
(2) Increasing the inlet water temperature not only results in an improved outlet water temperature, but also enhances heat exchange, thus promoting the total heat transfer coefficient.
(3) The total heat transfer coefficient is greater when the gas cooler is positioned vertically compared to horizontally placed in the parallel flow.
(4) Different heat transfer correlations were summarized to evaluate their accuracy when applied to engineering design. Zhang’s correlation was found to be more precise, and the calculation methods for pressure drop were summarized. The maximum error in calculating the CO_2_ pressure drop was found to be 4 kPa. However, due to the smaller viscosity of CO_2_, the calculation error for CO_2_ pressure drop was relatively large.

## Figures and Tables

**Table 1:** Important dimensions of the gas cooler.

Overall Dimensions	
Gas cooler length	200 mm
Gas cooler width	200 mm
Gas cooler height	86 mm
Gas cooler single later plate thickness	2 mm
**Carbon Dioxide Side**	
Carbon dioxide channel length	2.2 m
Carbon dioxide channel radius	1 mm
**Water Side**	
Elliptical channel length	2.2 m
Elliptical channel short semiaxis	1.3 mm
Elliptical channel long semiaxis	2.5 mm

**Table 2:** The information of the experimental equipment.

Equipment	Types	Models	Range	Accuracy
Compressor	Air-driven gas booster compressor	Haskel, AGD-32	25 MPa, 0~0.05 kg/s	-
CO_2_ flowmeter	Coriolis mass flowmeter	Sincerity, DMF-1-3A	0.0139~0.1389 kg/s	0.2%
Water flowmeter	electromagnetic flowmeter	YIHUA	0~0.3 kg/s	0.5%
Heater	Electric heater	HY-380-25kW	25 kW	-
Chiller	Air-cooled	XX-05A	15 kW	-
Differential pressure sensors	-	Yokogawa	0~200 kPa	0.075%
Pressure sensors	-	GAPT-I-H-0.25-25	0~25 MPa	0.25%
Temperature sensors	RTD	WZPB-230	0~100 °C; 0~150 °C	±0.2 °C

**Table 3:** Different heat transfer coefficient correlations of CO_2_.

Literatures	Correlations	Notes
Coons (1947) [2]	$\bar{Nu}=8.4{\left(\frac{mc_{p}}{kL}\right)}^{0.2}$	Laminar flow
	Nu¯=0.023Re0.8Prm	Turbulent flow *m* = 0.3 for cooling *m* = 0.4 for heating
McAdams (1954) [25]	Nu=0.0231.0+3.54d2RRe0.8Prm	*m* = 0.3 for cooling, *m* = 0.4 for heating
Baird (1957) [8]	Nu¯=CRe0.75Prm	*m* = 0.3 for fluid gaining heat, *m* = 0.4 for fluid losing heat C = 0.055, 4 < Pr < 10 and 9000 < Re < 78,000
Minton (1970) [26]	U=0.0231.0+3.54dDScpGRe−0.2Pr−2/3	Re>Recr
Buonopance and Troupe (1970) [10]	Nu¯=0.0235Re0.81Pr1/3	
	Nu¯=0.0111Re0.87Pr1/3	Parallel flow
Zhang (1988) [11]	Nu=0.0231.0+10.3dR3Re0.8Prm	*m* = 0.3 for cooling, *m* = 0.4 for heating
	Nu¯=0.10027Re0.682Prm	8000 < Re < 80,000 *m* = 0.3 for cooling, *m* = 0.4 for heating
Morimoto and hotta (1988) [27]	Nu¯=0.02391.0+5.54dRmRe0.806Pr0.268 $R_{m}=\frac{R_{\min}+R_{\max}}{2}$	
Inagaki(1998) [28]	Nu=0.78Re0.51Pr0.3	6000 < Re < 22,000

**Table 4:** Experiment conditions.

Case	$G_{{\mathrm{co}}_{2}}$ (kg·m^−2^·s^−1^)	P (MPa)	$T_{water,in}$ (℃)
1	183	8	31
2	162	8	31
3	146	8	31
4	162	7.5	26
5	162	7.5	27
6	183	8.5	31

## Data Availability

Not applicable.

## Nomenclature

**LMTD**	logarithmic mean temperature difference
Q	heat exchange, kW
$\dot{m}$	mass flow rate, kg·s^−1^
H	enthalpy, kJ·kg^−1^
T	temperature, °C
P	pressure, MPa
U	total heat transfer coefficient, W·m^−2^·K^−1^
h	enthalpy, kJ·kg^−1^
A	heat exchange area, m^2^
Nu	Nusselt number
Re	Reynolds number
Pr	Prandtl number
d	diameter, mm
N	number of spiral turns
L	spiral channel length, m
G	mass flux, kg·m^−2^·s^−1^
k	constant
ν	velocity, m·s^−1^
f	friction factor
*Greek symbol*	
λ	thermal conductivity, W·m^−1^·K^−1^
μ	dynamic viscosity, Pa·s
ρ	density, kg·m^−3^
*Subscription*	
*b*	bulk
*w*	wall
*i*	inlet
*o*	outlet
